# Intensity and mode of *Lindera melissifolia* reproduction are affected by flooding and light availability

**DOI:** 10.1002/ece3.8037

**Published:** 2021-08-22

**Authors:** Theodor D. Leininger, Emile S. Gardiner, Brian Roy Lockhart, Nathan M. Schiff, Alphus Dan Wilson, Margaret S. Devall, Paul B. Hamel, Kristina F. Connor

**Affiliations:** ^1^ USDA Forest Service Southern Research Station Center for Bottomland Hardwoods Research Stoneville MS USA; ^2^ Formerly with USDA Forest Service Southern Research Station Center for Bottomland Hardwoods Research Stoneville MS USA

**Keywords:** asexual reproduction, light availability, *Lindera melissifolia*, pondberry, sexual reproduction, soil flooding

## Abstract

We studied the impact of flooding and light availability gradients on sexual and asexual reproduction in *Lindera melissifolia* (Walt.) Blume, an endangered shrub found in floodplain forests of the Mississippi Alluvial Valley (MAV), USA. A water impoundment facility was used to control the duration of soil flooding (0, 45, or 90 days), and shade houses were used to control light availability (high = 72%, intermediate = 33%, or low = 2% of ambient light) received by *L. melissifolia* established on native soil of the MAV. Sexual reproductive intensity, as measured by inflorescence bud count, fruit set, and drupe production, was greatest in the absence of soil flooding. Ninety days of soil flooding in the year prior to anthesis decreased inflorescence bud counts, and 45 days of soil flooding in the year of anthesis lessened fruit set and drupe production. Inflorescence bud development was the greatest in environments of intermediate light, decreased in high‐light environments, and was absent in low light environments. But low fruit set diminished drupe production in intermediate light environments as compared to high light environments. Asexual reproduction, as measured by development of new ramets, was greatest in the absence of soil flooding and where plants were grown in high or intermediate light. Plants exhibited plasticity in reproductive mode such that soil flooding increased the relative importance of asexual reproduction. The high light environment was most favorable to sexual reproduction, and reproductive mode transitioned to exclusively asexual in the low light environment. Our results raise several implications important to active management for the conservation of this imperiled plant.

## Graphical abstract

We studied the impact of flooding and light availability gradients on sexual and asexual reproduction in *Lindera melissifolia* (Walt.) Blume, an endangered shrub found in floodplain forests of the Mississippi Alluvial Valley, USA. Plants exhibited plasticity in reproductive mode such that soil flooding increased the relative importance of asexual reproduction. The high light environment was most favorable to sexual reproduction, and reproductive mode transitioned to exclusively asexual in the low light environment.

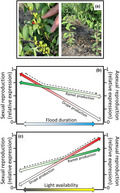

## 1 INTRODUCTION

Reproduction by clonal plants has two basic modes—asexual reproduction is accomplished through vegetative formation of a clone, while sexual reproduction is accomplished through seed formation. Asexual reproduction in clonal woody plants is commonly initiated through the development of rhizomes, root suckers, layered branches, or lignotubers (Jeník, 1994). This reproductive mode increases genet size, facilitates resource capture, and maintains genet longevity often under suboptimal or heterogeneous environmental conditions (Hutchings & Wijesinghe, 2008; de Witte & Stöcklin, 2010). Sexual reproduction in clonal woody plants enables dispersal of new genets thereby promoting genetic variation in existing populations and establishment of new populations (Eriksson, 1992; Kanno & Seiwa, 2004; Stöcklin & Winkler, 2004). While benefits of each reproductive mode are tempered by a range of ecological and energetic costs, the ability to reproduce via two different modes provides clonal plants with reproductive plasticity (Gardner & Mangel, 1999; Herben et al., 2015; Lei, 2010).

Investigations in temperate forests demonstrate that plasticity in expression of reproductive mode by clonal woody plants is driven by changes in components of the forest environment across space and time (Bunnell, 1990; Hewitt, 2020; Hosaka et al., 2008; Kanno & Seiwa, 2004; Moola & Vasseur, 2009; Salter et al., 2010). Variations in the availability of light, soil moisture, and other biotic and abiotic resources can affect resource acquisition, which then influences resource allocation patterns of a plant. Photosynthate allocation to components of vegetative or sexual reproduction supports the primary reproductive mode expressed by the plant in response to its environment. In an old‐growth Japanese beech (*Fagus crenata* Blume) forest, for example, flowering by the understory shrub *Hydrangea paniculata* Sieb. was limited to disturbed habitats of forest gaps (Kanno & Seiwa, 2004). However, this shrub reproduced almost exclusively through layering where canopy disturbance was lacking (Kanno & Seiwa, 2004). Other studies of temperate forest species found that expression of the two reproductive modes is affected by resource gradients, sexual reproduction especially is affected by light availability (Bunnell, 1990; Eckerter et al., 2019; Hosaka et al., 2008).

Understory environments of floodplain forests throughout the temperate zone are characteristically heterogeneous (Hall & Harcombe, 1998; Küẞner, 2003; Suzuki et al., 2002). Stand development and canopy disturbance dynamics control understory light regimes while alluvial processes active on the floodplain determine variability in edaphic and hydrologic components of the environment. Soil flooding, sediment accretion, and substrate erosion interplay with stand development and canopy disturbance agents, such as windstorms, ice storms, or insect and pathogen outbreaks, resulting in complex and often interacting gradients of resource availability. Several authors have linked species occurrence and growth in floodplain forest understories to plant stress tolerance and resource availability along gradients of flooding and light availability (Battaglia & Sharitz, 2006; Küßner, 2003; Lin et al., 2004; Sakai et al., 1999). Less is known about how these disturbance‐mediated environmental gradients in floodplain forests regulate expression of reproductive mode by understory woody plants capable of reproductive plasticity. However, evidence suggests that the relative expression of sexual and asexual reproduction is not controlled solely by one factor, such as light availability (Hosaka et al., 2008).

*Lindera melissifolia* (Walt.) Blume, commonly known as pondberry, is a dioecious, rhizomatous, and deciduous shrub in the Lauraceae (Devall et al., 2001). It is endangered but found in wet forests of the southeastern USA, namely, in Alabama, Arkansas, Georgia, Mississippi, Missouri, North Carolina, and South Carolina (Echt et al., 2011). In the floodplain forests of the Mississippi Alluvial Valley (MAV), *L. melissifolia* forms predominately single‐sex colonies in the understory of mixed, deciduous broadleaves (Hawkins et al., 2009; Wright, 1994). The recovery plan developed for *L. melissifolia* following its listing as an endangered species identified soil moisture and light intensity as key environmental factors to consider and understand better regarding the sustainable management of the species (USFWS, 1993). We wanted to know how these two environmental factors affect plasticity in expression of the reproductive modes in *L. melissifolia*.

**FIGURE 1:**
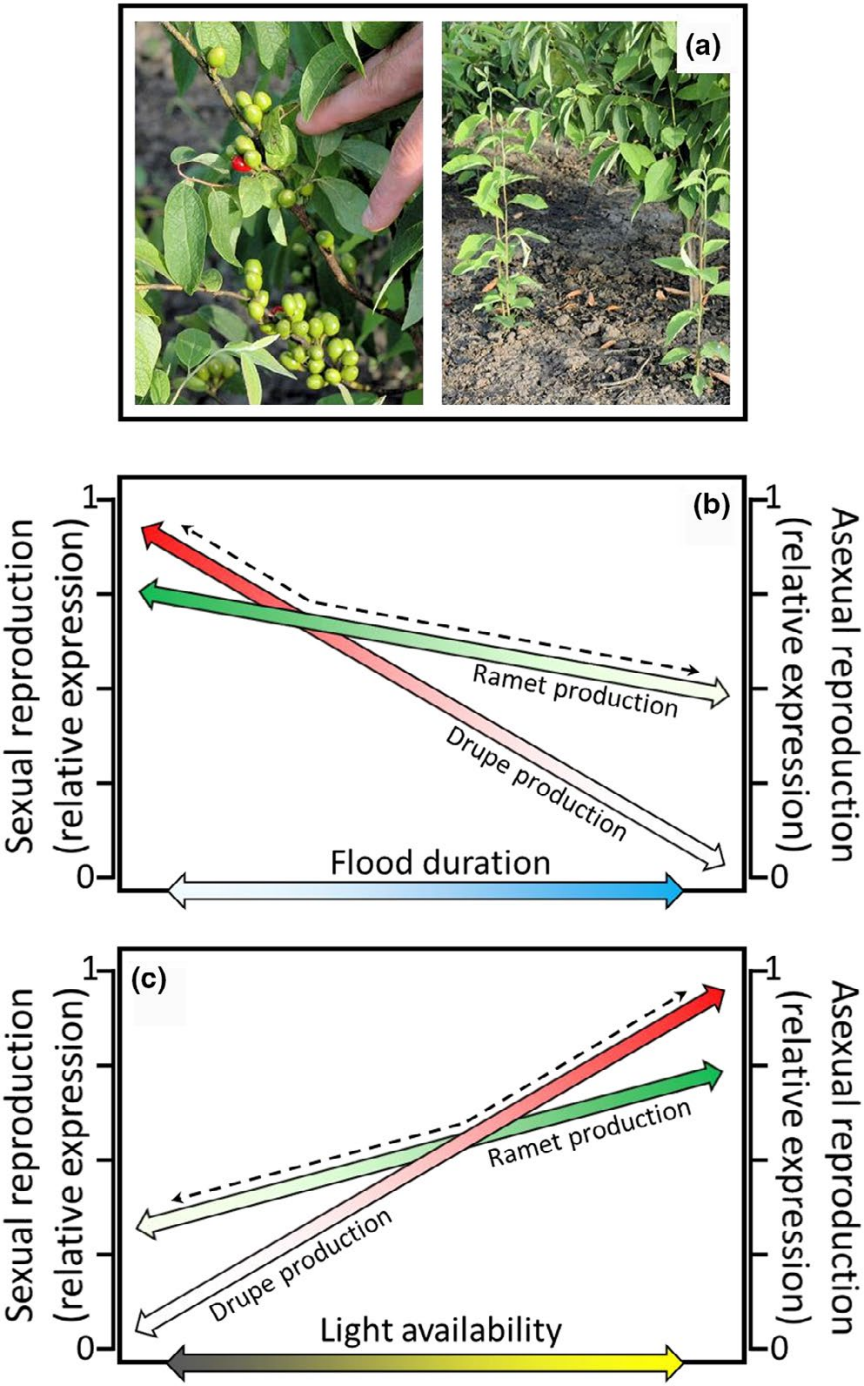
(a) The two reproductive modes (sexual and asexual) of *Lindera melissifolia*. Conceptual models of how (b) soil flooding and (c) light availability influence the reproductive modes of female *L. melissifolia*. Dashed lines parallel the *L. melissifolia* reproductive mode hypothesized to be favored along the gradient of each factor

To address our question, we established an experiment to investigate the effects of soil flooding and light availability on drupe (sexual reproduction) and ramet (asexual reproduction) production (Figure 1a) by female *L. melissifolia*. Our hypotheses were drawn from conceptual models of *L. melissifolia* reproductive modes relative to soil flooding (Figure 1b) and light availability (Figure 1c). Field observations indicate that *L. melissifolia* anthesis begins prior to leaf out when soil moisture typically is high, and in many instances when soils are flooded. However, we anticipated inflorescence bud formation, fruit set, and drupe production to sharply decrease as the duration of soil flooding increased into the growing season because of disruptions to physiological processes associated with anaerobiosis (Lockhart et al., 2017) (Figure 1b). We also expected ramet production to be limited by soil flooding, but to a lesser extent than drupe production. This is because rhizome and new ramet growth do not appear to occur when the soil is inundated, but likely resumes after floodwater recedes and an aerobic soil environment prevails (Lockhart et al., 2013). Thus, we hypothesized that *L. melissifolia* would favor sexual reproduction when grown in soil not subject to flooding but favor asexual reproduction as the duration of soil flooding increased into the growing season (Figure 1b, dashed line). We also predicted that inflorescence bud formation, fruit set, and drupe production by *L. melissifolia* would be greatest in a relatively high light environment, and these would decline as light availability decreased because of limitations to photosynthate production (Lockhart et al., 2017) (Figure 1c). Ramet production was also expected to decline with decreasing light availability, but more gradually than drupe production (Lockhart et al., 2013). Accordingly, we hypothesized that *L. melissifolia* would favor sexual reproduction when grown in high light environments but favor asexual reproduction in environments of relatively low light availability (Figure 1c, dashed line).

## 2 MATERIALS AND METHODS

### 2.1 Study species

As noted above, *L. melissifolia* anthesis begins prior to leaf expansion, typically in late February or early March for MAV populations (Hawkins et al., 2010). The insect‐pollinated, yellow flowers that are 5‐ to 6‐mm wide with 2‐mm long tepals usually arise in clusters of 3 from umbellate, axillary inflorescences. Drupes mature in August and September to an average of 11‐mm long and contain a 6‐mm‐long seed (Connor et al., 2007; Hawkins et al., 2010). Field observations indicate that *L. melissifolia* can produce large crops of drupes, but Devall et al. (2001) noted that seedling establishment rarely has been observed. *L. melissifolia* reproduces asexually by generating rhizomes that give rise to ramets (Wright, 1990). Information on *L. melissifolia* ramet biology is sparse, but Wright (1994) suggested asexual reproduction appears to be the dominant form of *L. melissifolia* regeneration. Also, of consequence to *L. melissifolia* regeneration, populations are reported to be male biased, with male to female colony ratios in the MAV ranging from 7:1 to 19:1 (Hawkins et al., 2007; Wright, 1994).

### 2.2 Study site

Our study was conducted in a 6‐ha impoundment network called the Flooding Research Facility (FRF) on the Theodore Roosevelt National Wildlife Refuge Complex, Sharkey County, Mississippi, USA (32°58′N, 90°44′W, 30 m elevation) (Lockhart et al., 2006). This experimental site is within 5 km of natural *L. melissifolia* colonies growing on the USDA Forest Service's Delta National Forest. The site lies in a humid, subtropical region of the temperate zone—average daily temperature at the FRF is 17.3°C with a range from 27.3°C in July to 5.6°C in January, and precipitation averages 1,350 mm annually (WorldClimate, 2008). Soil within the FRF is Sharkey clay (very‐fine, smectitic, thermic Chromic Epiaquerts), it is alluvial in origin and a predominant soil series in the MAV. The FRF consists of 12, 0.4‐ha, rectangular impoundments that can be independently flooded or drained to create and control replicates of experimental hydroperiods. Lockhart et al. (2006) present more detailed information regarding design and operation of the FRF.

### 2.3 Plant material and establishment

Planting stock for this experiment consisted of 20 *L. melissifolia* genotypes that we collected in the MAV and replicated with tissue culture techniques as described in Hawkins et al. (2007). Rooted cuttings of each genotype were container‐grown for about 11 months in greenhouses after which stem length averaged 21.6 ± 0.3 cm (mean ± one standard error) and basal diameter averaged 1.8 ± 0.01 mm. We delineated three, 19.2‐m by 7.2‐m plots in each of the 12 FRF impoundments (36 total plots). For each of the 36 plots, we randomly selected 96, single‐stemmed plants and transplanted them on a 1.2‐m by 1.2‐m spacing in April 2005 (3,456 total plants). Female genotypes comprised 54% of the experimental population. Transplants were acclimated to the field environment during the remainder of 2005—we replaced those that did not survive through May 2005. Weeds were eliminated in all plots for the duration of the experiment by manual cultivation supplemented with directed herbicide applications.

### 2.4 Experimental factors

Our experimental design included three levels each of two factors, soil flooding and light availability, used to provide gradients of environmental conditions that could result from two different disturbance types (inundation and forest canopy damage) prevalent in floodplain forests across the range of *L. melissifolia*. Soil flooding was imposed by randomly assigning one of three flooding regimes to each of the 12 impoundments. The three regimes represent a linear increase in flooding duration: 0 days of soil flooding, 45 consecutive days of soil flooding, or 90 consecutive days of soil flooding. Flooding of designated impoundments began the year after planting on 1 March 2006 and was repeated on 1 March 2007 such that impoundments were flooded for assigned intervals in two consecutive growing seasons. Water used to flood impoundments was primarily rainfall captured and stored in an adjacent reservoir, but some ground water was used to supplement stored rainfall, as needed. Flood‐water depth was maintained near 12 cm above the soil surface in 2006 and 19 cm above the soil surface in 2007 when experimental plants were taller. Impoundments were drained at the end of each scheduled flood, and ambient rainfall was the only source of soil moisture during nonflooded periods.

We constructed three shade houses in each impoundment to control light availability in a fashion representative of a range of forest canopy cover. A shade house consisted of a 25.6‐m long by 7.3‐m wide by 2.4‐m tall wooden frame covered with neutral density shade cloth (PAK Unlimited, Inc., Cornelia, Georgia, USA). Shade houses were built over areas in each impoundment noted above as plots. Each shade house in an impoundment was randomly assigned a relatively “high,” “intermediate,” or “low” level of light availability. We used 30% shade cloth to provide high light, 63% shade cloth to provide intermediate light, and 95% shade cloth to provide low light. Actual light availability measured in shade houses (Lockhart et al., 2013) differed from shade cloth ratings such that plots received an average of 72%, 33%, or 2% of available photosynthetically active radiation for the high, intermediate, and low levels, respectively. Shade house construction was completed prior to transplanting, so that light availability assignments were in place during field acclimation of plants in 2005.

### 2.5 Measurements

We measured variables of sexual and asexual reproduction on all female *L. melissifolia* plants during 2007. The soil flooding treatment had been implemented 1 year prior, and the light availability treatment had been implemented 2 years prior to our measurements. Thus, our measurements are reflective of plant responses following 2 episodes of controlled soil flooding and 3 years of controlled light availability. Inflorescence buds, easily distinguished from shoot buds by their location and relatively large size, were counted on plants in January and February 2007. In September 2007, drupes were fully mature when we collected them and placed them in cold storage at 2°C for further processing. A random sub‐sample of 100 drupes, including pedicels, was drawn to quantify their fresh and dry weights (g). Length and basal diameter of all stems for each plant were measured and noted by type (original or ramet) at the end of the 2007 growing season. These measurements were used to calculate plant and ramet growth from similar measurements collected in 2006. We also destructively sampled shoot mass from six randomly selected plants in each plot (216 total plants). The shoots, which were harvested in September 2007, were separated into leaf, stem, drupe, and drupe pedicle tissues that were oven dried at 70°C then weighed to the nearest 0.01 g.

### 2.6 Experimental design and analyses

A completely randomized, split‐plot design was used to evaluate the effects of soil flooding and light availability on *L. melissifolia* reproductive modes. The 12 impoundments at the FRF accounted for four replicates of the three flooding regimes and represented the whole‐plot factor in the experimental design. The three, 19.2‐m by 7.2‐m plots (within shade houses) in each impoundment received one replicate of a light availability level and represented the split‐plot factor in the experimental design. Analyses were conducted on plot means using PROC GLIMMIX with an adjustment in the error term for the whole‐plot factor (SAS 9.4, SAS Institute, Inc.). PROC UNIVARIATE was used to test data normality for each response variable, and residual errors were normalized with Box–Cox, natural log, or square root transformations where appropriate prior to the PROC GLIMMIX analyses. Significance was accepted at ∝ = 0.05, and we used the least significant difference (LSD) test to separate significant treatment effect means. When a soil flooding and light availability interaction was significant, separation of soil flooding level means was conducted by light availability level, and separation of light availability level means was conducted by soil flooding level.

Response variables measured on female *L. melissifolia* and analyzed in this experiment are listed and briefly defined in Table 1. Fruit set for each plant that flowered was calculated by dividing drupes (no.) by inflorescence buds (no.) multiplied by 3—note that *L. melissifolia* inflorescence buds typically produce 3 flowers each. The number (no.) of ramets produced in the current study year (2007) was calculated as the difference between 2007 and 2006 counts, and the smallest ramets were assumed to have developed in 2007. We defined reproductive intensity (no.) as drupes (sexual reproduction) or ramets (asexual reproduction) counted on a plant. Reproductive intensity ratio (no.) was calculated by dividing drupe counts by ramet counts for each plant. We used stepwise regression on stem mass, length, and basal diameter data collected from the harvested shoots to build a model that predicted plant shoot mass (excluding drupes and pedicels) from stem basal diameter. This model was applied to measures of stem basal diameter to estimate ramet mass produced in 2007 by each plant. The average dry weight of sub‐sampled drupes and pedicels (0.29 g) was multiplied by drupe counts to estimate the total dry weight of drupes and pedicels produced by each plant. Reproductive mass (g) was defined as the total weight of drupes and their pedicels, or total weight of ramets for a plant, and reproductive mass ratio (g) was calculated by dividing drupe and pedicle mass by ramet mass.

**TABLE 1:** Variables analyzed to evaluate effects of soil flooding and light availability on reproductive mode of female *Lindera melissifolia*

Variable^1^	Description
Inflorescence buds^2^	Count per plant (no.)
Inflorescence buds per unit stem length^2^	Count per cm of total plant stem length (no./cm)
Drupes^2^	Count per plant (no.) ‐ This variable represents the sexual reproductive intensity of a plant
Drupes per unit stem length^2^	Count per cm of total plant stem length (no./cm)
Fruit set^2^	[drupes/[inflorescence buds*3]]*100 (%)
Ramets^3^	Count per plant (no.) ‐ This variable represents the asexual reproductive intensity of a plant
Reproductive intensity ratio	Drupes/ramets (no.)
Reproductive mass	Drupe + pedicel mass (g) for sexual reproduction Ramet mass (g) for asexual reproduction
Reproductive mass ratio	Drupe + pedicel mass/ramet mass (g)

^1^ All variables were measured at the plant level, and plant‐level measurements were used to compute plot‐level means for analysis.

^2^ This variable was only analyzed on plants that produced inflorescence buds.

^3^ Only those produced during the current study year (2007).

## 3 RESULTS

### 3.1 Inflorescence bud production and fruit set

Mean inflorescence bud production by *L. melissifolia* ranged between 0 and 485 per plant showing substantial variation relative to soil flooding and light availability (Table 2). Plants receiving 90 days of flooding produced 33% fewer buds than plants receiving 45 or 0 days of flooding (Table 2). The extended flood also limited inflorescence bud production per unit of stem to about 77% of that observed for plants receiving the 45‐day flood (Table 2).

**TABLE 2:** The average number of inflorescence buds and inflorescence buds per unit stem length for female *Lindera melissifolia* plants relative to soil flooding and light availability in 2007, Sharkey County, Mississippi, USA

Treatment	Level	Inflorescence buds^1^, ^2^ (no.)	Buds per length^3^ (no./cm)
Soil flooding	0 days	468.2 ± 41.5 a	1.34 ± 0.05 ab
	45 days	466.8 ± 35.1 a	1.50 ± 0.09 a
	90 days	313.5 ± 35.9 b	1.15 ± 0.08 b
Light availability	High	346.9 ± 29.9 b	1.41 ± 0.07 a
	Intermediate	485.5 ± 31.5 a	1.26 ± 0.07 a
	Low^4^	0	0

^1^ Values are means ± standard error, and letters in a column by treatment indicate differences at *p* ≤.05.

^2^ Test statistics: soil flooding × light availability (*F*_(2,9)_ = 0.49, *p* = .62); soil flooding (*F*_(2,9)_ = 6.90, *p* = .01); light availability (*F*_(1,9)_ = 28.69, *p* = .0005).

^3^ Test statistics: soil flooding × light availability (*F*_(2,9)_ = 0.06, *p* = .94); soil flooding (*F*_(2,9)_ = 4.49, *p* = .04); light availability (*F*_(1,9)_ = 3.60, *p* = .09).

^4^ Plants receiving low light availability did not produce inflorescence buds, so they were excluded from this analysis.

Inflorescence bud production by *L. melissifolia* was greatest when plants were grown under intermediate light (Table 2), with plants raised under this light level producing 40% more buds than those raised under high light. But, inflorescence bud counts relative to stem length were equivalent for these two light levels. *L. melissifolia* grown under low light did not develop inflorescence buds (Table 2).

**FIGURE 2:**
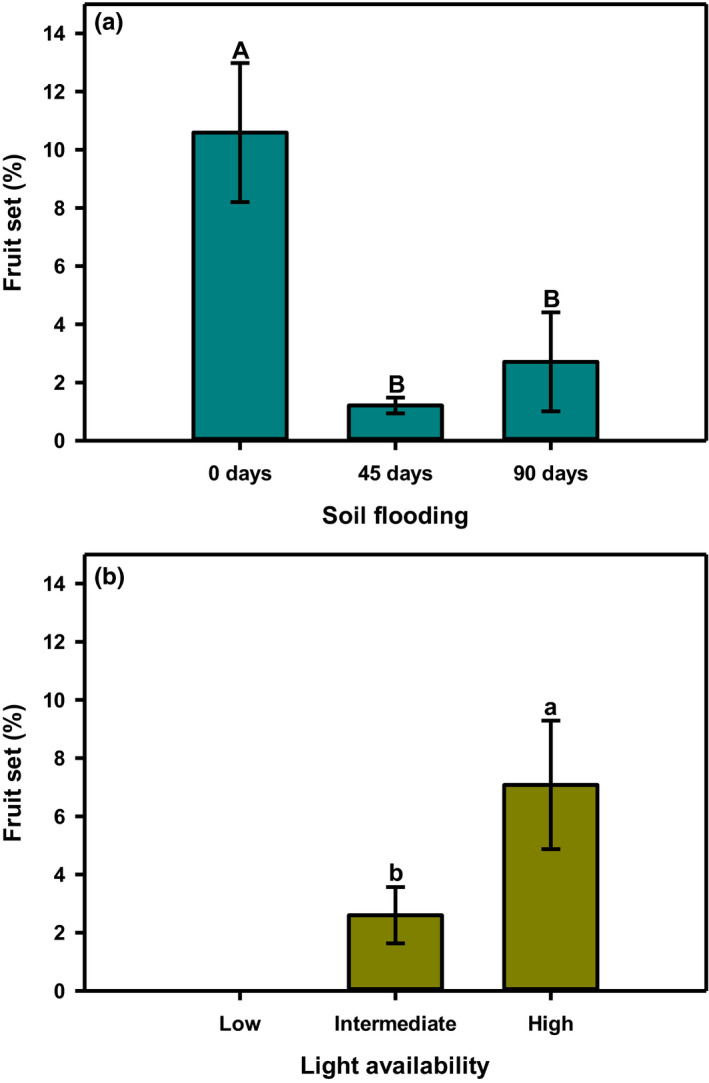
Fruit set for female *Lindera melissifolia* plants relative to soil flooding and light availability in 2007, Sharkey County, Mississippi, USA. Mean ± standard error with letters indicating difference at *p* ≤ .05. Plants receiving low light availability did not produce inflorescence buds, so they were excluded from this analysis

Between 1% and 11% of all *L. melissifolia* flowers set fruit, and the likelihood of fruit set varied considerably because of soil flooding and light availability (Figure 2). Relative to soil flooding, the highest fruit set (10.6%) was observed for plants raised free of flooding. Fruit set averaged about 2% when plants received soil flooding, and it did not differ between the 45‐ and 90‐day floods (Figure 2a). We observed an increase in fruit set with increasing light availability (Figure 2b). About 7% of flowers produced a fruit when plants were grown under high light. This is a 170% greater fruit set than for plants receiving intermediate light. As mentioned above, plants raised under low light did not flower, so fruit set was not a possibility.

### 3.2 Reproductive intensity

Collectively, *L. melissifolia* plants produced over 54,000 drupes in 2007. Soil flooding and light availability acted independently to influence the average number of drupes produced per plant (Table 3). In respect to soil flooding, plants raised in plots assigned 0 days of flooding yielded the greatest number of drupes. Drupe production decreased by at least 85% among plants receiving the 45‐day or 90‐day floods. An environment of high light availability supported the greatest yield of drupes per plant (Table 3). Plants raised beneath intermediate light produced 43% fewer drupes than those receiving high light, but intermediate light supported substantial drupe production as compared to the low light environment (Table 3).

**TABLE 3:** The average sexual (drupes) and asexual (ramets) reproductive intensity (no.) for female *Lindera melissifolia* plants relative to soil flooding and light availability in 2007, Sharkey County, Mississippi, USA

Variable	Light availability	Soil flooding^1^			Mean
		0 days	45 days	90 days	
Drupes^2^	High	147.4 ± 21.8	23.2 ± 4.6	31.4 ± 21.3	67.4 ± 19.5 a
	Intermediate	97.6 ± 24.2	8.9 ± 1.8	9.0 ± 2.6	38.5 ± 14.6 b
	Low^3^	0	0	0	0
	Mean	122.5 ± 17.8 A	16.1 ± 3.6 B	20.2 ± 10.8 B	
Ramets^4^	High	11.7 ± 0.9 Aa	7.3 ± 0.5 Ba	6.9 ± 1.2 Ba	8.6 ± 0.8
	Intermediate	11.1 ± 1.0 Aa	6.6 ± 0.8 Ba	7.4 ± 1.2 Ba	8.3 ± 0.8
	Low	0.8 ± 0.1 Ab	0.7 ± 0.1 Ab	0.3 ± 0.1 Ab	0.6 ± 0.1
	Mean	7.8 ± 1.6	4.9 ± 0.9	4.8 ± 1.1	

^1^ Values are means ± standard error. Capital letters indicate differences within a row for soil flooding means, lower case letters indicate differences in a column for light availability means at *p* ≤ .05.

^2^ Test statistics: soil flooding x light availability (*F*_(2,9)_ = 1.11, *p* = .37); soil flooding (*F*_(2,9)_ = 16.57, *p* = .001); light availability (*F*_(1,9)_ = 8.34, *p* = .02).

^3^ Plants receiving low light availability did not produce inflorescence buds, so they were excluded from this analysis.

^4^ Test statistics: soil flooding x light availability (*F*_(4,18)_ = 3.90, *p* = .02); soil flooding (*F*_(2,9)_ = 9.76, *p* = .006); light availability (*F*_(2,18)_ = 126.86, *p* < .0001).

We observed development of more than 9,400 new ramets in 2007. Soil flooding and light availability interacted to influence the number of ramets produced per plant (Table 3). Plants that were grown in the absence of soil flooding (0 days of soil flooding) and with either high or intermediate light produced an average of more than 11 new ramets. Floods of either 45 or 90 days, coupled with these same light environments, reduced the number of ramets produced by plants at least 39%. Low light availability limited development of new ramets to an average of less than 1 per plant for all levels of soil flooding (Table 3).

### 3.3 Reproductive intensity ratio

**FIGURE 3:**
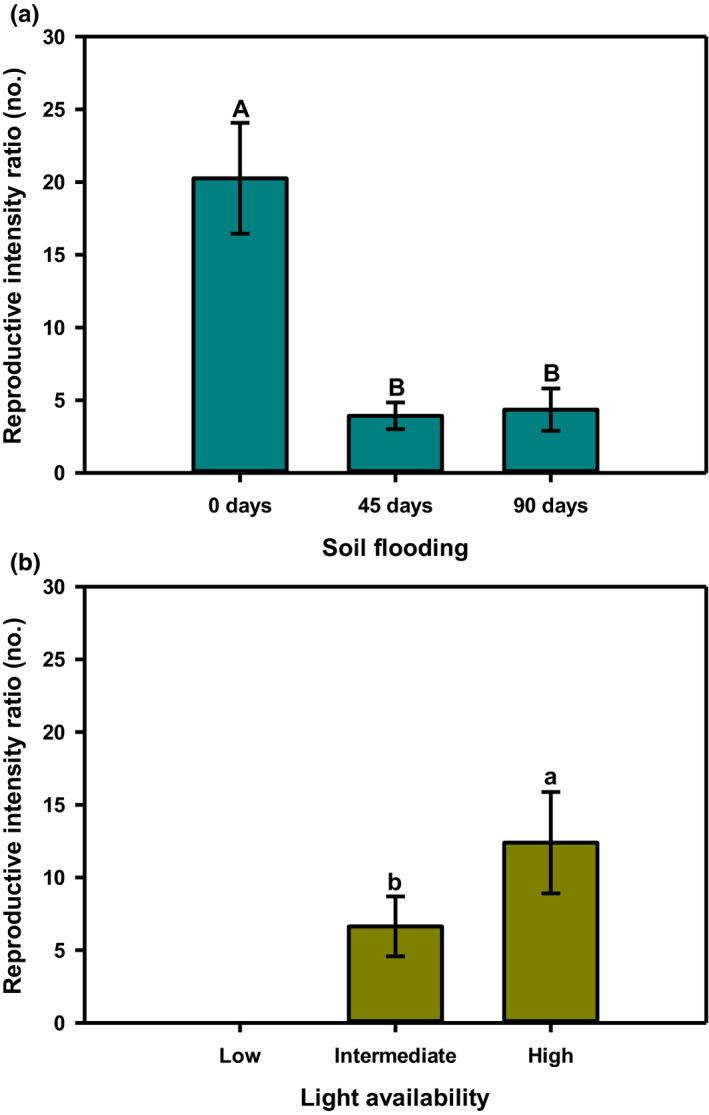
Reproductive intensity ratio by (a) soil flooding and (b) light availability for female *Lindera melissifolia* plants relative to soil flooding and light availability in 2007, Sharkey County, Mississippi, USA. Mean ± standard error with letters indicating difference at *p* ≤ .05. Plants receiving low light availability did not produce inflorescence buds, so they were excluded from this analysis

The reproductive intensity ratio of *L. melissifolia* was generally greater than 1, the exception being plants established under low light (Figure 3). Mean values of reproductive intensity ratio relative to soil flooding levels ranged between 20:1 and 4:1 (Figure 3a). Plants raised in the absence of flooding (0 days soil flooding) showed the highest reproductive intensity ratio, soil flooding for either 45 or 90 days reduced the ratio by 79% (Figure 3a). The reproductive intensity ratio relative to light availability was greatest (12:1) under high light, decreased 46% for plants receiving intermediate light, and was 0 for plants receiving low light (Figure 3b).

### 3.4 Reproductive mass

Soil flooding and light availability independently affected total drupe mass of *L. melissifolia* (Table 4). Soil flooding for either 45 or 90 days reduced accumulation of drupe mass per plant by about 85% as compared to 0 days of soil flooding (Table 4). Total drupe mass per plant averaged 74% higher among plants receiving high light versus those receiving intermediate light regardless of soil flooding.

**TABLE 4:** The average sexual (drupes) and asexual (ramets) reproductive mass (g) for female *Lindera melissifolia* plants relative to soil flooding and light availability in 2007, Sharkey County, Mississippi, USA

Variable	Light availability	Soil flooding^1^			Mean
		0 days	45 days	90 days	
Drupes^2^	High	42.8 ± 6.3	6.7 ± 1.3	9.1 ± 6.2	19.5 ± 5.6 a
	Intermediate	28.3 ± 7.0	2.6 ± 0.5	2.6 ± 0.8	11.2 ± 4.2 b
	Low^3^	0	0	0	0
	Mean	35.5 ± 5.2 A	4.7 ± 1.0 B	5.9 ± 3.1 B	
Ramets^4^	High	356.9 ± 17.9 Aa	235.2 ± 12.5 Ba	197.6 ± 39.3 Ba	263.2 ± 24.6
	Intermediate	392.5 ± 38.9 Aa	227.9 ± 40.3 Ba	261.6 ± 46.0 Ba	294.0 ± 30.6
	Low	5.9 ± 1.5 Ab	2.3 ± 0.3 Ab	2.6 ± 0.5 Ab	3.6 ± 0.7
	Mean	251.8 ± 54.2	155.2 ± 34.9	153.9 ± 37.9	

^1^ Values are means ± standard error. Capital letters indicate differences within a row for soil flooding means, lower case letters indicate differences in a column for light availability means at *p* ≤ .05.

^2^ Test statistics: soil flooding × light availability (*F*_(2,9)_ = 1.11, *p* = .37); soil flooding (*F*_(2,9)_ = 16.57, *p* = .001); light availability (*F*_(1,9)_ = 8.34, *p* =.02).

^3^ Plants receiving low light availability did not produce inflorescence buds, so they were excluded from this analysis.

^4^ Test statistics: soil flooding × light availability (*F*_(4,18)_ = 3.88, *p* = .02); soil flooding (*F*_(2,9)_ = 11.19, *p* = .004); light availability (*F*_(2,18)_ = 55.7, *p* < .0001).

Soil flooding and light availability interacted to influence total ramet mass of *L. melissifolia* (Table 4). Accumulation of ramet mass was greatest, averaging about 375g, for plants raised in the absence of flooding (0 days soil flooding) and in environments of high or intermediate light availability. Soil flooding for 45 or 90 days reduced this mass by 38% (Table 4). Accumulation of ramet mass by *L. melissifolia* was least under low light availability, and soil flooding did not impact this response (Table 4).

### 3.5 Reproductive mass ratio

**FIGURE 4:**
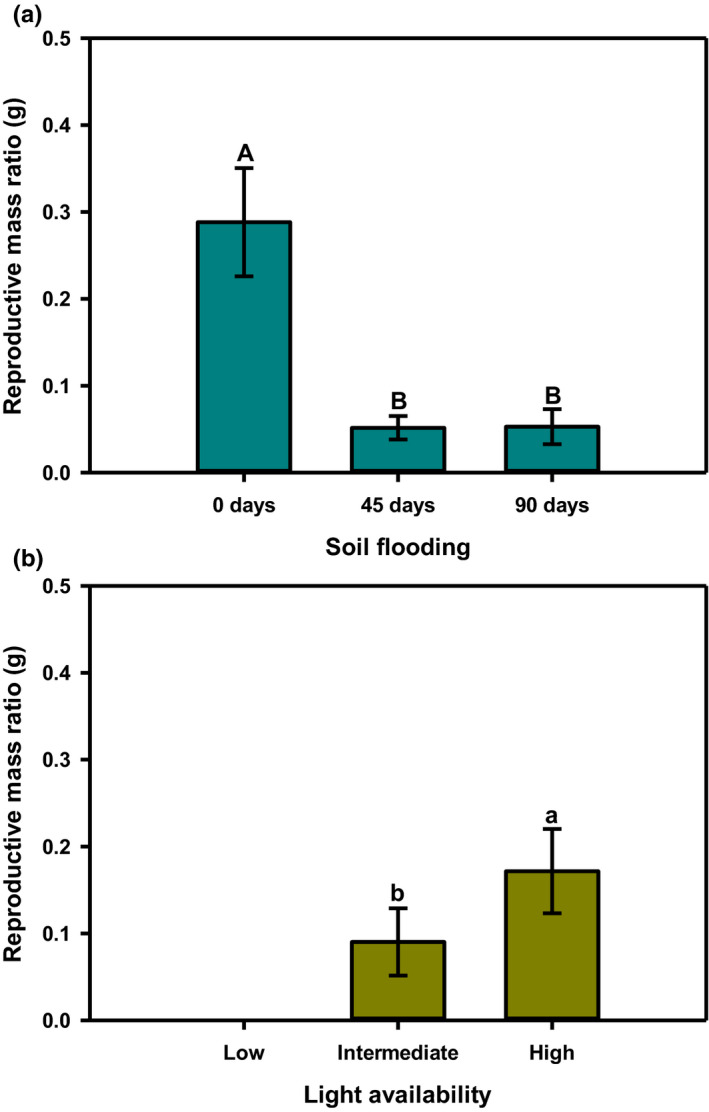
Reproductive mass ratio by (a) soil flooding and (b) light availability for female *Lindera melissifolia* plants relative to soil flooding and light availability in 2007, Sharkey County, Mississippi, USA. Mean ± standard error with letters indicating difference at *p* ≤ .05. Plants receiving low light availability did not produce inflorescence buds, so they were excluded from this analysis

*L. melissifolia* showed reproductive mass ratios less than 1 for all levels of soil flooding and light availability (Figure 4). The highest reproductive mass ratio (about 1:3.5) was observed for plants grown in the absence of flooding (0 days soil flooding), regardless of light availability (Figure 4a). Soil flooding for 45 or 90 days reduced the ratio to about 1:19 (Figure 4a). Across all soil flooding regimes, plants receiving high light showed reproductive mass ratios of about 1:6, those receiving intermediate light showed a ratio of about 1:11, and those receiving low light showed a ratio of 0 (Figure 4b).

## 4 DISCUSSION

Understanding processes of regeneration is fundamental to conservation of threatened and endangered plant species. *L. melissifolia*, an endangered shrub found in floodplain habitats of the Mississippi Alluvial Valley, is capable of two modes of reproduction, sexual and asexual. Expression of reproductive mode appears plastic among *L. melissifolia* colonies—sites occupied by *L. melissifolia* colonies occur at different points along gradients of environmental factors that drive this plasticity. Recent research has advanced our understanding of the physiological mechanisms and their genetic foundations that initiate and sustain processes of sexual and asexual reproduction, and how environmental cues play primary roles in expression of floral and vegetative growth (Conti, 2017; Schneider et al., 2019; de Wit et al., 2016). The amount and quality of light, particularly, are known to activate genes responsible for synthesis of flowering hormones and floral growth (Conti, 2017; Schneider et al., 2019; de Wit et al., 2016). Conversely, stress resulting from some environmental factors will activate genes that repress or inhibit floral and vegetative growth (Kazan & Lyons, 2016). Basic knowledge of these processes, from a species conservation perspective, enriches our understanding of the complexities of plant reproduction, and underpins research that supports regenerating targeted species in heterogeneous environments. Aside from these advances, the need remains for field research at plant and population levels that informs management for conservation. The current study reveals insights into reproductive intensity and plasticity in expression of the reproductive modes in *L. melissifolia* relative to soil flooding and light availability, two prominent environmental factors in its floodplain habitat.

### 4.1 Sexual reproduction

We hypothesized that soil flooding would reduce sexual reproductive intensity of *L. melissifolia*, and this effect would be most pronounced where flood duration was greatest. Our results are that soil flooding affected *L. melissifolia* sexual reproduction by reducing inflorescence bud count, inflorescence bud count per unit of stem, fruit set, and drupe production and mass. Flooding prior to, and in the year of, anthesis differentially affected inflorescence bud development and fruit set, respectively. Ninety days of soil flooding in 2006 decreased inflorescence bud counts prior to 2007 anthesis. Soil flooding for 45 and 90 days in the year of anthesis (2007) lessened fruit set and drupe production. Though soil flooding imposed in consecutive years showed differing effects on variables of sexual reproduction, reproductive intensity, as measured by drupe production, did not show a greater decline when flooding extended beyond 45 days.

Investigations into flooding effects on reproductive intensity in clonal woody plants of temperate floodplain forests are limited. In a recent study, Greet et al. (2020) observed a decreased number of flower buds, flowers, and capsules produced by *Eucalyptus camphora* R.T. Baker subject to prolonged flooding in a temperate swamp forest of southeastern Australia. The tropical floodplain tree, *Melaleuca quinquenervia* (Cav.) S.T. Blake, showed arrested seed development and viability in response to permanent flooding (Rayachhetry et al., 1998). Flooding studies conducted on plants occurring outside of floodplains also are informative of negative effects on sexual reproduction. Twenty‐five days of flooding reduced flower bud formation and fruit set by *Vaccinium virgatum* Ait., a shrub native to the southeastern USA (Crane & Davies, 1985). Likewise, four months of soil flooding reduced the number of inflorescence buds and flowers per inflorescence in *V*. *corymbosum* L., another shrub species common throughout eastern North America (Abbott & Gough, 1987). Our results provide clear evidence that intensity of sexual reproduction in *L. melissifolia* is lessened by soil flooding of timing and duration typical of its MAV floodplain habitat.

We hypothesized that light availability would distinctly affect sexual reproduction in *L. melissifolia* leading to diminished reproductive intensity in relatively low light environments and strong reproductive intensity in relatively high light environments. Light availability altered *L. melissifolia* sexual reproduction through changes in inflorescence bud count, inflorescence bud count per unit of stem, fruit set, and drupe production and mass. Inflorescence bud development, which was determined in the 2006 growing season, was greatest in environments of intermediate light, decreased in high light environments, and did not occur in low light environments. The number of buds produced per stem length was similar in high and intermediate light environments, indicating that the greater inflorescence bud production under intermediate light resulted from plants having more reproductive stem length in that environment (Lockhart et al., 2013). Though plants in intermediate light environments produced a relatively high number of inflorescence buds in 2006, a low fruit set in 2007 diminished drupe production and mass compared to that of plants in high light environments. Consistent with our hypothesis, we observed a positive relationship between light availability and reproductive intensity as measured by drupe production—reproductive intensity rose incrementally with increasing light availability.

Light availability exerts strong influence on sexual reproduction for many clonal, woody plants in temperate understories (Hosaka et al., 2008; Kanno & Seiwa, 2004; Roper et al., 1995). *V. myrtillus* L. produced a greater number of flower buds, flowers, and fruit in the relatively high light environment of forest gaps compared to closed‐canopy locations in southern Germany (Eckerter et al., 2019). In southeastern Pennsylvania, the proportion of *Lindera benzoin* (L.) Blume flowers that initiated fruit was the same on control and shaded branches but significantly fewer fruit matured on shaded branches (Niesenbaum, 1993). Flower abundance in *Sambucus racemosa* L. in the Pacific Northwest, USA was greater in gaps than under intact forest canopies (Wender et al., 2004). Sexual reproduction requires greater photosynthate availability than asexual reproduction (Holsinger, 2000). Kanno and Seiwa (2004) studied the Asian shrub *H. paniculata* and noted a proliferation in the number of flowering individuals with increasing light availability from canopy gap formation. For older gaps showing canopy closure, Kanno and Seiwa (2004) speculated that light was too limiting for plants to produce enough photosynthates for sexual reproduction. Likewise, Hosaka et al. (2008) reported that the light environment of gaps was more conducive to flowering than was the light environment of closed‐canopy forests for *Asimina triloba* (L.) Dunal, an understory tree of eastern North America. In contrast, Unks et al. (2014), who studied *L. melissifolia* at 2 field sites in North Carolina, USA, were unable to establish a correlation between light transmittance through the overstory and the number of flowering stems in colonies. Our results demonstrate the strong effect of light availability on intensity of sexual reproduction in *L. melissifolia*. Light availability characteristic of the understory in closed‐canopy broadleaf forests of the MAV were too low to support sexual reproduction by this species. Light availabilities characteristic of partial overstories supported substantial sexual reproduction with the environment representative of relatively large canopy openings providing for the greatest reproductive intensity.

### 4.2 Asexual reproduction

We predicted soil flooding would limit asexual reproduction of *L. melissifolia* by inhibiting ramet production in parallel with flood duration. Likewise, we predicted an increasing limitation on asexual reproduction with decreasing light availability. In contrast to our findings for sexual reproduction, the effects of soil flooding on *L. melissifolia* asexual reproduction were conditioned by light availability, that is, the two effects were not independent, they interacted. Ramet production and mass were greatest in the absence of soil flooding and where plants were grown in high or intermediate light. For these same light environments, soil flooding of 45 or 90 days led to diminished numbers and mass of new ramets. Low light supported minimal development of new ramets and mass regardless of flooding regime. Thus, neither soil flooding nor light availability affected asexual reproduction of *L. melissifolia* in strict accord with our hypotheses—the predicted linear response of ramet production to each of these effects was not observed.

*L. melissifolia* produced ramets throughout the growing season, but no new ramets appeared from plants during active flooding. This observation suggests that the soil flooding effect was simply a shortening of the amount of time during the growing season conducive to asexual reproduction. In other words, soil flooding suspended the *L. melissifolia* growing season. Plant growth and ramet development proceeded when floodwater was removed. But, the effect of soil flooding does not appear this simple because we did not observe an incremental decrease in ramet production when flooding was extended from 45 to 90 days. We are unable to speculate as to why this effect did not progress beyond that observed for the 45‐day flood.

Very little has been documented about flooding effects on asexual reproduction in woody, clonal plants of temperate floodplain forests (Yang & Kim, 2016). However, research conducted on perennial plants from other systems indicates that species responses may diverge based on their predominant form of asexual reproduction and the nature of hydrologic regimes in their habitats. We observed that the stress of soil flooding reduced ramet development in *L. melissifolia*, but some floodplain species may amplify asexual reproduction in response to apparent flood‐related stress. For example, Chong et al. (2013) demonstrated that epicormic sprouting of layered branches and stems in *M. leucadendra* (L.) L. led to genet persistence and growth in riverine environments of relatively high magnitude flooding, but this species did not exhibit similar clonal growth in small, tributary environments of relatively low magnitude flooding. Also, maximal tiller production by the perennial grass, *Arundinella hirta* (Thunb.) Koidz., occurred at low topographic positions of highest flood duration on a central China floodplain (Zeng et al., 2006).

*L. melissifolia* ramet development relative to light availability patterned somewhat consistently with other temperate woody plants that possess the ability to reproduce via rhizomes or root sprouting. Most of these clonal plants can generate new ramets across a wide range of light environments, but ramet development often peaks in high‐light environments more supportive of plant vigor (Kawamura & Takeda, 2002, 2008; Kowarik, 1995). However, Hosaka et al. (2008), who studied *A. triloba* in temperate broadleaf forests in Maryland, USA, reported that ramet recruitment did not correlate with light availability beneath closed canopy versus canopy gap locations. Also, clonal plants that rely on other forms of asexual reproduction, such as layering or fragmentation, may show higher rates of ramet development in relatively low‐light environments where genet persistence is critical (Kanno & Seiwa, 2004).

### 4.3 Plasticity in reproductive mode

Plants capable of sexual and asexual reproduction often exhibit reproductive plasticity by favoring one reproductive mode over the other in response to biotic or abiotic environmental factors (Loehle, 1987; Yang & Kim, 2016). We hypothesized that *L. melissifolia* would exhibit plasticity in expression of reproductive mode along gradients of soil flooding and light availability. No soil flooding was expected to favor sexual reproduction, but reproductive mode would transition to favor asexual reproduction with increasing duration of soil flooding. Likewise, a high light environment was expected to favor sexual reproduction, but reproductive mode would transition to favor asexual reproduction with decreasing light availability.

Our results for reproductive intensity ratio and reproductive mass ratio demonstrate impact of soil flooding on the relative expression of reproductive mode by *L. melissifolia*. A sharp reduction in drupe production and mass occurred with flooding regardless of light availability. Ramet production and mass also declined with flooding, but the effect was not as sharp as with drupes. As to be expected, reproductive intensity ratio and reproductive mass ratio illustrate opposing results for the relative importance of a reproductive mode. For *L. melissifolia*, reproductive intensity ratio appears biased towards sexual reproduction, and reproductive mass ratio appears biased toward asexual reproduction. Nevertheless, both indices reveal that the relative importance of sexual reproduction was greatest in the absence of soil flooding, and this reproductive mode lost considerable importance with 45 or 90 days of soil flooding. Thus, it appears that soil flooding invoked a plastic response in *L. melissifolia* reproductive mode by increasing the importance of asexual reproduction while decreasing the importance of sexual reproduction. Other authors reporting a similar observation associate asexual reproduction with the capacity to recover from disturbance and enhanced genet persistence in harsh floodplain environments (Chong et al., 2013; Zeng et al., 2006). Though flooding altered the relative importance of each reproductive mode, we did not find evidence to support our hypothesis that soil flooding would prompt a transition from favoring one reproductive mode to the other. These results may be the first to document the effect of soil flooding on expression of reproductive mode in an understory shrub endemic to temperate floodplain forest habitats.

A gradient of light availability also elicited a plastic response in *L. melissifolia* reproductive mode. Reproductive intensity ratio and reproductive mass ratio each illustrate that the relative importance of sexual reproduction was greatest in the high light environment, and this reproductive mode lost importance with decreasing light availability. Asexual reproduction increased in relative importance with decreasing light availability. In fact, we observed a transition in reproductive mode to exclusively asexual reproduction in the low light environment, and this observation, to some extent, supports our hypothesis.

A transition in reproductive mode across an environmental gradient of light availability (or surrogates of light availability) has been reported for other understory shrubs of temperate forests including *Gaultheria shallon* Pursh in Canada (Bunnell, 1990) and *H. paniculata* Sieb. in Japan (Kanno & Seiwa, 2004). Along an environmental gradient of light, sexual reproduction is often associated with relatively high light availability because processes of flowering and fruit development are energetically costly requiring a highly functional photosynthetic system (Holsinger, 2000). As we observed for *L. melissifolia*, intensity of sexual reproduction tends to decrease as shading increases, and asexual reproduction becomes the more prominent reproductive mode in clonal plants (Kanno & Seiwa, 2004; Kawamura & Takeda, 2002). In this research, *L. melissifolia* was capable only of asexual reproduction when grown in the low light environment. Though minimal, ramet development in this environment enabled plants to increase photosynthetic surface area and capture growing space, both of which foster genet persistence in habitats of suboptimal light (Kanno & Seiwa, 2004; Yang & Kim, 2016).

### 4.4 Implications to conservation

Our research is the first to examine reproductive biology of *L. melissifolia* relative to environmental factors prominent in its floodplain habitat. We discovered impacts to *L. melissifolia* sexual and asexual reproductive intensity and expression of plasticity in reproductive mode due to soil flooding. Flooding regimes, similar in seasonality, depth, and duration to natural flooding experienced by *L. melissifolia* colonies in the Mississippi Alluvial Valley, differentially lessened sexual and asexual reproductive intensity. This differential suggests that annual variation in floodplain inundation, to a large degree, regulates the relative output of each reproductive mode. Additionally, the intensity of sexual reproduction was determined through aggregated effects of two soil flooding events. Flooding in the year prior to anthesis negatively affected inflorescence bud development, and flooding in the year of anthesis negatively affected fruit set and drupe production. Management strategies targeting *L. melissifolia* conservation should allow for periodic failures in drupe production and/or years of minimal ramet development and colony expansion that result from the unpredictable nature of floodplain inundation. Fostering regeneration of this species will require vigilance in assessing floodplain inundation events, their impacts to inflorescence bud development, drupe production, and ramet development and growth, and flexibility in application of management practices to support and sustain developing cohorts, either seedlings or ramets, of reproduction.

We also demonstrate responses to sexual and asexual reproductive intensity and plasticity in reproductive mode of *L. melissifolia* along a gradient of light availability, that is, the light environment of a given *L. melissifolia* colony will differentially affect intensity and relative expression of reproductive mode. Both sexual and asexual reproductive intensity were maximal in environments of relatively high light. Low light availability inhibited flowering and was suboptimal for ramet development. The large number of drupes and ramets produced by shrubs receiving high or intermediate light shows the potential for *L. melissifolia* to reproduce sexually and asexually when provided with favorable light environments.

Light availability in understories of mature temperate forests varies spatially and temporally because of canopy gaps and the distribution of foliage among canopy layers (Canham et al., 1990; Runkle, 1982). In the absence of canopy gaps, light available in the understory of mature floodplain forests is minimal averaging <5% of full sunlight over the course of a day (Cunningham et al., 2011; Jenkins & Chambers, 1989). This is likely the case in floodplain forests of the MAV where *L. melissifolia* grows because these forests develop multi‐storied canopies that significantly reduce light transmission to the understory (Hawkins et al., 2009; Oliver et al., 2005). Forest stand structure can be managed to create light environments that promote *L. melissifolia* colony vigor and structures conducive to sexual and asexual reproduction.

Our summary of findings presented above highlights the ability of *L. melissifolia* to respond reproductively to favorable hydrologic and light environments. Still, an additional implication to conservation of this imperiled species may be drawn from our observations of its reproductive plasticity. Along with decreases in reproductive intensity, we report change in the relative importance of reproductive modes expressed by *L. melissifolia* along soil flooding and light availability gradients. Specifically, we observed an increase in the relative importance of asexual reproduction as soil flooding and low light availability limited sexual reproduction. This reproductive plasticity, which confers persistence in suboptimal habitats, is a trait of high importance to conservation management because it allows for flexibility in the timing and intensity of management activities. For example, management practices aimed at improving the light environment in habitats of multi‐storied, closed‐canopy floodplain forests could be implemented in methodical stages that sequentially reduce mid‐story and overstory canopy cover. Such an approach would enable the manager to monitor and react to the *L. melissifolia* response, as well as the response of competing vegetation or other factors that potentially impact *L. melissifolia* survival, growth, and reproduction.

Historical deforestation in the MAV—72% loss of forest cover (Gardiner, 2015)—has reduced greatly the availability of potential forest habitat for *L. melissifolia*. The current state of forest land cover in the MAV may lead managers toward active management to conserve this species. We contribute knowledge of *L. melissifolia* reproductive intensity and mode that is essential to the long‐term conservation of this species in floodplain forests of the MAV.

## CONFLICT OF INTEREST

None declared.

## AUTHOR CONTRIBUTIONS

**Theodor D. Leininger:** Conceptualization (equal); Funding acquisition (lead); Project administration (lead); Supervision (lead); Writing‐original draft (equal); Writing‐review & editing (equal). **Emile S. Gardiner:** Conceptualization (equal); Data curation (lead); Formal analysis (equal); Funding acquisition (equal); Investigation (equal); Methodology (equal); Supervision (equal); Visualization (lead); Writing‐original draft (equal); Writing‐review & editing (equal). **Brian Roy Lockhart:** Formal analysis (equal); Investigation (equal); Methodology (equal); Supervision (equal); Writing‐original draft (equal); Writing‐review & editing (equal). **Nathan M. Schiff:** Conceptualization (equal); Funding acquisition (equal); Writing‐review & editing (equal). **Alphus Dan Wilson:** Conceptualization (equal); Funding acquisition (equal); Writing‐review & editing (equal). **Margaret S. Devall:** Conceptualization (equal); Funding acquisition (equal); Writing‐review & editing (equal). **Paul B. Hamel:** Conceptualization (equal); Funding acquisition (equal); Writing‐review & editing (equal). **Kristina F. Connor:** Conceptualization (equal); Funding acquisition (equal); Writing‐review & editing (equal).

## Data Availability

Original data pertaining to this research are available on the Dryad Digital Repository: https://doi.org/10.5061/dryad.63xsj3v2x
